# Effects of the Environment on Developmental Plasticity and Infection Success of *Schistosoma* Parasites – An Epigenetic Perspective

**DOI:** 10.3389/fmicb.2019.01475

**Published:** 2019-07-09

**Authors:** Ronaldo de Carvalho Augusto, David Duval, Christoph Grunau

**Affiliations:** IHPE UMR 5244, University of Perpignan Via Domitia, CNRS, IFREMER, Perpignan, France

**Keywords:** schistosomiasis, environmental cues, inheritance systems, imune response, host-para site interactions

## Abstract

Evidence of how environmental cues affect the phenotypes of, and compatibility between *Schistosoma mansoni* and their hosts come from studies in environmental parasitology and research on host diet and chemotherapeutic treatment. Schistosomes deal with a multitude of signals from the water environment as well as cues that come from their hosts, particularly in response to molecules that serve to recognize and destroy them, i.e., those molecules that arise from their hosts’ immune systems. These interactions shape, not only the parasite’s morphology, metabolism and behavior in the short-term, but also their infection success and development into different stage-specific phenotypes later in their life cycle, through the modification of the parasite’s inheritance system. Developmental phenotypic plasticity of *S. mansoni* is based on epigenetic mechanisms which are also sensitive to environmental cues, but are poorly understood. Here, we argue that specific cues from the environment could lead to changes in parasite development and infectivity, and consequently, environmental signals that come from environmental control measures could be used to influence *S. mansoni* dynamics and transmission. This approach poses a challenge since epigenetic modification can lead to unexpected and undesired outcomes. However, we suggest that a better understanding of how environmental cues are interpreted by epigenome during schistosome development and host interactions could potentially be applied to control parasite’s virulence. We review evidence about the role of environmental cues on the phenotype of *S. mansoni* and the compatibility between this parasite and its intermediate and definitive hosts.

## Introduction

In the course of evolution, parasites improve their fitness as a result of the selection of traits which determine their relationships with hosts (Webster et al., 2007). Digenetic parasites which have multiple (as a rule obligatory) consecutive hosts face the additional problem that different hosts require specific parasite phenotypes plus free-living stages to transit between hosts. Furthermore, to develop, each stage must address and deal with a multitude of signals from the environment, such as temperature, pH, osmolarity and chemical compounds, and also signals that come from the host, in particular those that serve to recognize and destroy them, i.e., the immune system (Coustau et al., 2000; Cosseau et al., 2017; Sures et al., 2017). In this mini-review, the term “environment” will be used to refer to biotic and abiotic conditions that interact with the parasite at each stage of its life cycle. In *Schistosoma* (parasitic flatworms) this could be a freshwater environment, or the intermediate host or definitive host environment. This interaction shapes not only the parasite’s morphology, metabolism and behavior in the short-term, but also its development into different phenotypes over the whole of its life cycle, i.e., subsequent stages that were not directly exposed to that environment (Escobedo et al., 2005; Augusto et al., 2017). As discussed by Cosseau et al. (2017), the developmental and evolutionary trajectories of schistosomes are based on an inheritance system composed of at least three elements: (i) the genome *G* and (ii) the epigenome I, which are exposed to signals from (iii) the environment *E*. All three components interact to bring about the phenotype P in different time scales [the (*G* × *I*) × *E* =>*P* concept]. The dynamics of this system were recently demonstrated for the whole *S. mansoni* life cycle where epigenetic changes (histone methylations) are essential to generate phenotypically distinct stages (Roquis et al., 2018). Here, we briefly present a broad view of how environmental cues affect the phenotype and also the compatibility between *S. mansoni* and their hosts.

**FIGURE 1:**
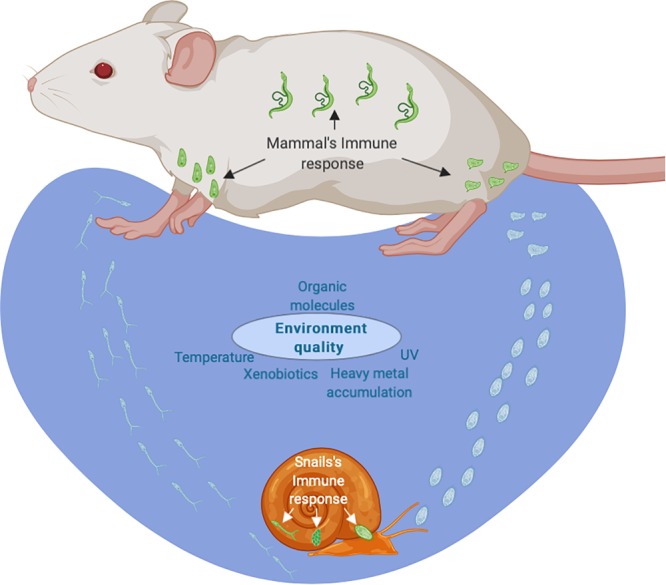
Life cycle of the human parasite *Schistosoma mansoni*. The life cycle starts when eggs (in green) are released by mammalian’s host and are affected by osmotic pressure in contact with freshwater (in blue) and deliver a free-swimming larva, the miracidium, that seek out an intermediate host, a freshwater snail of the *Biomphalaria* genus. Here, as a free-swimming larva, is the first time that the parasite is susceptible to an abiotic environment (in blue). After this first, miracidia have to penetrate the tegument of the snail host (in green) and transform into primary sporocysts while dealing with a sophisticated immune system with barrier functions in the epithelium, a cellular immune response and a humoral defense response. Cercariae (in green) are realized and it is the second time that schistosomes face water quality (in blue). Cercaria larva actively seek a definitive mammalian host (rodent, primate or human). After infection, schistosomules migrates through the venous environment to develop into adult parasites and to reproduce sexually while dealing with humoral and cellular immune responses. In blue – eggs and free-swimming stages under cues of the water environment. In green – parasitic stages under cues from the internal environment of the hosts.

Intestinal schistosomiasis is a chronic parasitic disease caused mainly by the trematode ***S. mansoni***. Around 67 million people are infected worldwide and hundreds of thousands remain exposed to the risk of parasitic infection by contact with infested water used for crop irrigation, for recreational or for domestic purposes (Jamison et al., 2006; Steinmann et al., 2006; Morgan et al., 2010). The parasite has a complex life cycle which involves two consecutive obligatory hosts and two transitions between these hosts as free-swimming larvae; in each step, a new environment interacts with the parasite (Figure 1). The interaction with each environment demands regulation of gene expression to meet the parasite’s biological needs and/or to allow for interaction with the host’s immune response (Jolly et al., 2007; Jeremias et al., 2017; Lu et al., 2017; Vasconcelos et al., 2017). The life cycle starts when eggs are released into freshwater and the change in osmotic pressure triggers release of a free-swimming larva, the miracidium, that seeks out an intermediate host, a freshwater snail of the ***Biomphalaria*** genus. Here, as a free-swimming larva the parasite is susceptible for the first time to an abiotic environment outside the vertebrate host, with different water temperatures or soluble compounds, that can affect directly and/or indirectly the parasite’s biology. After this first environmental experience, miracidia have to penetrate through the tegument of the snail host and transform into primary sporocysts, multiply asexually, form secondary sporocysts and produce hundreds of cercariae while dealing with the snail’s immune system (Pinaud et al., 2016). Cercariae, a second type of free-swimming larvae, actively seek a definitive mammalian host (usually rodent, primate or human). It is the second time that schistosomes face water quality issues and again are vulnerable to freshwater pollutants such as pesticides, molluscicides and heavy metals, which can affect growth and development in the short or long term (King and Highashi, 1992; Liang et al., 2010; Augusto et al., 2017; Sures et al., 2017). Direct effects were observed, for instance, with non-toxic concentrations of silver nitrate that reduce cercarial infectivity by inhibition of lipid-induced penetration but do not affect the worm’s development after subcutaneous injection (King and Highashi, 1992). Other developmental effects are triggered, for example, by the molluscicide ***Euphorbia milii*** latex that does not affect cercarial infectivity, but which does lead to developmental changes inside the definitive host (Augusto et al., 2017). In addition, differential susceptibility of male and female worms to pollutants has been described, with possible epidemiological implications (Liang et al., 2010; Lamberton et al., 2017). Differential male and female cercarial susceptibility to Praziquantel (PZQ), the only anthelmintic drug widely applied, is still not entirely understood but might lead to mating bias in field populations in areas where mass drug administration is intense (Liang et al., 2010). Unfortunately, to our knowledge, little to no work has been done to evaluate the effects of different water soluble compounds on field populations so far. After infection, schistosomules migrate through the venous environment of the vertebrate host to develop into adult parasites and to reproduce sexually. Here they are exposed to a new host environment and must deal with humoral and cellular (adaptive) immune responses (summarized in section ***“What do we know about the influence of the environment on the interaction with the definitive vertebrate host?”***).

## What Do We Know About the Influence of the Environment on the Interaction With the Intermediate Snail Host?

Freshwater is the immediate environment for the intermediate snail host. In laboratory settings, *S. mansoni* infections in *Biomphalaria* spp. snails are commonly measured using snails grown in clean dechlorinated water, for which the chemical and biological composition can be different from water encountered by snails in the field. Indeed, freshwater snails can occupy different aquatic environments with varying degree of flow, pollution, and turbidity (Kloos et al., 2004). Also, many laboratory studies have defined the ecology of the snail by assessing the effects of salinity, pH, water depth, and temperature on snail physiology (Jurberg et al., 1987; Eveland and Haseeb, 2011; Kalinda et al., 2017). These parameters might influence the compatibility between parasite and snail, but as yet, experimental results are lacking. Other environmental cues that might impact host and parasite include: the presence of different contaminants such as pesticides, molluscicides, heavy metals or endocrine disruptors (Iqbal and Sinha, 2011; Augusto et al., 2015; Sures et al., 2017). Most ecotoxicological approaches have focused on the toxicity of these pollutants to uninfected or infected snails, using them as bioindicators (Bianco et al., 2014; Fahmy et al., 2014; Mostafa et al., 2014; Tallarico et al., 2014; Habib et al., 2016). The parasite’s intramolluscan development may also be affected by the type of feed used for snail breeding or rearing (Thompson et al., 1991; Fried et al., 2001). For example, although this observation is under debate, the development time for transformation of infective cercariae could be delayed with a high lipid diet (Thompson et al., 1991; Fried et al., 2001). Also, the number of cercariae produced from each successful miracidial infection can be significantly increased with protein-rich foods (Coles, 1973). Unsurprisingly, most of these studies were designed to determine the toxicity of water contaminants for the snails, and only a few studies have investigated the parasite’s physiological changes and/or changes to molecular pathways which could impact host/parasite compatibility. Environmental perturbations can, of course, affect the immunological and physiological parameters of *Biomphalaria* spp. snails, and thus, change the relationship between the host and the parasite in one way or another (Table 1).

**Table 1:** Effect of environmental cues on biology and molecular pathways of freshwater intermediate host.

Environmental determinants	Biological effect	Molecular pathways	References
Euphorbia milii latex	Molluscicidal activity, physiological stress and altered reproduction	nd	Mello-Silva et al., 2010, 2011; Augusto et al., 2015
Endocrine disruptors (Bisphenol A and Phthalate)	Increased oviposition and reproduction traits	nd	Iqbal and Sinha, 2011
Cadmium	Lethal effect, locomotion alteration, acquisition of thermal tolerance, Diminution of egg hatching and Increased parasite sensitivity	HSP70 gene expression +	Salice and Roesijadi, 2002; Habib et al., 2016; da Silva Cantinha et al., 2017
Manganese	Lethal effect	nd	Habib et al., 2016
Zinc oxide nanoparticles	Molluscicidal activity	NO concentration + GST protein – GST, CAT and SOD activities –	Fahmy et al., 2014
Chromium	Lethal toxicity and an embryonic developmental effect	nd	Tallarico et al., 2014
Azinphos-methyl (organophosphorus insecticide)	Lethal effect	Carboxylesterases activity –	Bianco et al., 2014
Diazinon and Profenfos (organophosphorus compound)	Lethal effect	SOD, CAT, GR, TrxR, and SDH activities – Lipid peroxidation +	Bakry et al., 2016
Paraquat (herbicide)	Lethal effect	SOD activity – Lipid peroxidation +	Cochón et al., 2007
Butachlor and Fluazifop-p-butyl (herbicide)	Lethal effect	Acid and alkaline phosphatases concentration +	Tantawy, 2002
Chlorine and Huwa-san desinfectant	Lethal effect	SOD and GST activities –	Tantawy, 2002
Glyphosate	Lethal effect	Total hemocytes + Phagocytic activity + DNA damage +	Mohamed, 2011
Niclosamide and derivatives	Lethal effect	NOS, AChE and LDH activities – Hemoglobin, NOS, SOD and FREP4 expression – HSP20, HSP40 and HSP70 expression + CYP and GST expression +	Zhang et al., 2015; He et al., 2017
Circadian cycle disruption	Host susceptibility	Total hemocytes –	Waissel et al., 1999; Steinauer and Bonner, 2012

*Nd, non detected studies; (++), increase; (-), decrease; NO, nitric oxide; NOS, nitric oxide synthase; HSP, heat shock proteins; GST, Glutathione S-transferases; CAT, catalase; SOD, superoxide dismutase; GR, glutathione reductase; TrxR, thioredoxin reductase; SDH, sorbitol dehydrogenase; AChE, acetylcholinesterase; LDH, Lactate dehydrogenase; CYP, cytochrome P450; FREP4, fibrinogen related protein 4.*

It is important to highlight here that snail-schistosome redox dynamics play a crucial role in compatibility, based on a complex interplay between host defenses and the parasite’s strategies to circumvent the immune response (Mitta et al., 2017). Reactive oxygen species (ROS) are one of the main immune effector molecules involved in the snail’s attempts to stop the parasite’s development (Hahn et al., 2000). The major form of ROS involved in sporocyst blocking is hydrogen peroxide (H_2_O_2_), a compound produced by the snail’s hemocytes (Hahn et al., 2001). Interestingly, susceptible snails release less H_2_O_2_, indicating they might have lower SOD activity after parasite infection (Bender et al., 2005). Comparative genetic analyses between susceptible (compatible) and resistant (incompatible) snails support an association between compatibility and allelic variation and/or expression at the SOD locus (Blouin et al., 2013; Tennessen et al., 2015). To protect the sporocyst from deleterious oxidative effects, several antioxidant enzymes or scavengers are produced by the sporocyst itself (e.g., GST, glutathione peroxidase, peroxiredoxin, thioredoxin) (Guillou et al., 2007; Mourão et al., 2009; Wu et al., 2009). The reduction of parasite antioxidant activity by an antifungal agent decreases its snail infectivity (Moné et al., 2010). Moreover, experiments with different host-parasite combinations have shown that parasites displaying high levels of ROS scavenger production have higher infection success, and conversely, snails with low oxidative capability are more susceptible (Moné et al., 2011). In response to the dynamics of environmental cues, parasites show adaptive plasticity for the ROS scavenger production trait (Moné et al., 2011). This could be due to epigenetic changes (Li et al., 2018).

The *S. mansoni*-snail interaction is characterized by a phenomenon called “compatibility polymorphism,” meaning that some parasite-host combinations lead to infection success (they are compatible) and others do not (they are incompatible). *S. mansoni* mucin gene (*Sm*PoMuc) is a conserved family of polymorphic mucins which have been shown to be key markers for compatibility polymorphisms observed between different strains of *S. mansoni* and *B. glabrata* (Roger et al., 2008; Perrin et al., 2013; Fneich et al., 2016). Expression of *Sm*PoMuc is associated with histone modifications, such as trimethylation or acetylation of histone 3 lysine 9 (H3K9me3, H3K9ac) (Fneich et al., 2016). Different enrichment profiles in *Sm*PoMuc promoters have been observed between the compatible and incompatible strains (Perrin et al., 2013). Treatment with inhibitors of histone modifying enzymes changed the expression of these *Sm*PoMuc phenotypic variants in *S. mansoni* and increased parasite compatibility with the intermediate reference host (Fneich et al., 2016). Another study addressed the influence of the snail host environment on frequency of epimutations that occurred in the parasite during interactions with *B. glabrata*. The impact of two host environments (an allopatric vs. a sympatric snail host) on different histone markers, including H3K4me3, H3K27me3, H3K27ac, and H4K20me1, was studied in cercariae emerging from the two host environments and on the resulting subsequent adult stages (Roquis et al., 2015). The authors found three types of epimutations: genotype-dependent, environment-dependent, and random epimutations. While most environmentally induced epimutations appear to be ephemeral in *S. mansoni*, epimutations that are passed through the germ line can arise through paramutations (Roquis et al., 2015). Paramutations are interactions between the two alleles of a locus, where one allele induces heritable changes in the other allele (reviewed in Chandler, 2007). Hybridization of a compatible and an incompatible (*vis-a-vis* a reference snail) *S. mansoni* strain led to heritable histone H3K9 acetylation and methylation changes in the above-mentioned *Sm*PoMuc, and was associated with increased infection success (compatibility) (Fneich et al., 2016). This goes in line with the idea that epigenetic modifications are a way to produce phenotypic plasticity and to survive in a changing environment (Hu and Barrett, 2017).

In summary, it is therefore indispensable to include measures of environmental (“ecotoxicological”) parameters, and to investigate the environmentally mediated epigenetic components, when evaluating snail-schistosome compatibility in the field. The interaction between the parasite, the environment, and the host can influence infection success and the risk of transmission.

## What Do We Know About the Influence of the Environment on the Interaction With the Definitive Vertebrate Host?

It is interesting to know that before infection, even brief contact with soluble pollutants can trigger changes on the free-swimming cercaria, and these changes can be inherited from one parasite stage to another. Ultimately, disease dynamics and host morbidity can be affected by epigenetic changes. We recently described that short exposure to a plant extract used as molluscicide (*Euphorbia milii* latex), at low doses, does not affect cercarial survival or infectivity, but does trigger not only a change in morphology, metabolic pathways, and fitness of the adult worms, but also the size of hepatic granulomas in the definitive host, an important clinical feature (Augusto et al., 2017).

Once inside its definitive host, variations of the parasite phenotype can be induced by certain intra-host cues (e.g., gene expression perturbations, host diet, drugs, and metabolic syndromes), which affect the parasite’s growth and development (Jolly et al., 2007; Thornhill et al., 2009; Roquis et al., 2015). The parasite’s surface, mainly the male tegument, has a particular importance. It releases several classes of antigens that interact with host antibodies and T cells (Jankovic et al., 1999). Details on immunology of human schistosomiasis were reviewed recently (Colley and Secor, 2014). Briefly, from the first moments after cercarial infection to the end of worm maturation in the blood stream, Th1-type immune responses against schistosomulae result in noticeable increase in certain cytokines (TNFα, IL1α, IL1β, and IL6) as well as Signal Transducers, Activators of Transcription 1 (STAT1), and IFNγ (Burke et al., 2009, 2010; Sanchez et al., 2017). However, once adult worms start depositing eggs around 6 weeks after initial infection, a dramatic shift to a Th2-type immune response ensues. Here, specific egg antigens promote several different classes of cytokines (IL4, IL5, IL10, IL13, and IL33), T regulatory cells, B cells, antibodies and anti-idiotypic responses; complex immunomodulatory mechanisms result in liver fibrosis and hepatosplenic disease but also are thought to have a host tissue protective function (Colley et al., 1999; Fairfax et al., 2012; Colley and Secor, 2014). Through molecular mimicry, adult schistosomes are able to avoid the host’s immune system, possibly through acquiring host antigens and incorporating them into their own surface (Keating et al., 2006; Jiz et al., 2009; Colley and Secor, 2014). Currently, PZQ is the main schistosomicidal compound used to treat the disease in humans, but in the past, other drugs such as Oxamniquine and Hycanthone were also used (Rosi et al., 1965). These chemical compounds effect deformations, such as wrinkling, erosion and loss of tubers, on the parasite’s surface (Shuhua et al., 2000; Manneck et al., 2010). When adult worms are exposed to PZQ, for instance, a progressive contraction of the longitudinal musculature is associated with significant influx in Ca^2+^, resulting in damage to the parasite’s surface (Gnanasekar et al., 2009; Pinto-Almeida et al., 2016). This goes in line with the finding that effectiveness of schistosomicidal compounds depends on establishment of sufficient surface damage to allow the host’s immune system to recognize the parasite as non-self (Brindley and Sher, 1987; Fallon et al., 1992; Doenhoff et al., 2008). Adult worms have long life expectancies (Colley et al., 2014), and it is well accepted that a large part of the mechanism allowing this may be due to the parasite’s tegument, which goes through a constant renewal process in the outer syncytium zone thanks to schistosome stem cells (neoblasts) (Collins et al., 2013). Epigenetic processes, based on DNA methylation machinery, maintain the proliferative capacity of schistosome neoblasts (Geyer et al., 2018b). Recently, it was demonstrated that even a partial depletion of DNA methylation machinery (based on RNA interference suppression of *S. mansoni* methyl-CpG-binding, *Sm*MBD2/3, and chromobox protein, *Sm*CBX) significantly reduces neoblast proliferation and egg production, and changes the parasite phenotype (Geyer et al., 2018b). This is particularly important because egg production impacts both human pathology and disease transmission. Furthermore, due to a growing understanding that schistosome development is regulated by epigenetic processes, a certain number of studies have been conducted to characterize important molecules (i.e., histone modifying enzymes) (Pierce et al., 2011, 2012; Cabezas-Cruz et al., 2014; Carneiro et al., 2014; Marek et al., 2015; Cosseau et al., 2017; Geyer et al., 2018a,b; Roquis et al., 2018). Specifically, changes in chromatin structure are observed during adult worm maturation inside the definitive host; likewise, sex-specific gene expression profiles can be observed throughout this process (Picard et al., 2016; Roquis et al., 2018).

Epigenetic processes provide a wealth of potential therapeutic targets for the development of novel therapies against schistosomiasis. The impact of drugs on the schistosome epigenome are mainly studied through dose-response trials, often carried out using *in vitro* approaches, whereas, studies on the impact of drugs on the schistosome epigenomics in realistic (non-laboratory) situations are still lacking. Since histones and histone modifications are conserved throughout the eukaryotes, many histone methyltransferase enzyme inhibitors have been used to understand the role of post-translation histone modifications in schistosomes (Cabezas-Cruz et al., 2014; Ballante et al., 2017; Padalino et al., 2018; Pereira et al., 2018; Roquis et al., 2018). Recently, Padalino et al. (2018) used a histone demethylase, Lysine Specific Demethylase 1 (SmLSD1, Smp_150560), *in vitro*, and found significant impacts on adult worm motility, reproduction rate, and phenotype. Drugs that disrupt epigenetic processes or inhibit the enzymes involved could offer novel therapeutics for controlling schistosomiasis. Interestingly, laboratory-induced, Hycanthone-resistant parasites present distinct chromatin structure following post-translation histone modifications: H3K4me3, H3K9me3/ac and H3K27me3 (Roquis et al., 2014). Even though the resistance phenotype might not be heritable (this was not investigated), transient improvements in survival might be sufficient to ensure higher reproductive success of the epigenetically modified individuals. To date, studies evaluating dose-response effects are more frequent than studies that reflect a systemic view of environmental cues and genetic and non-genetic inheritance in the life cycle and transmission of *S. mansoni*. Understanding the myriad ways in which environmental cues drive the schistosome life cycle should be helpful to explain geographical differences observed in parasite biology, distribution, spread, and morbidity, and might improve the effectiveness of field control approaches.

## Future Directions

Antihelminthic drugs are a relatively new experience (evolutionarily speaking) for the parasite. Cues that result from host nutrition, environmental quality, or even psychoactive substances ingested by the definitive host, such as tobacco or alcohol, are much older, but investigations on these latter topics are missing and should be undertaken. Analogies are evident in other parasite systems. For example, human daily ethanol ingestion has a positive association with frequency of *Strongyloides stercoralis* infection (Marques et al., 2010); chronic alcohol ingestion significantly reduces granuloma and hepatic fibrous tissue in mice infected with *S. mansoni* (Orrego et al., 1981; Castro et al., 1993); and a high-fat diet has a prominent effect on the course of chronic schistosomiasis mansoni in mice (Alencar et al., 2009). Modern molecular techniques are needed for better characterization of this phenomenon. While a direct (maybe toxic) effect of alcoholism in the human host might not be surprising, our group has suggested an additional rationale concerning the possible functional (and evolutionary) link between diet, drug consumption and schistosome snail infection (Fneich et al., 2016). In our model, changes in environmental cues would trigger an epigenetic switch between bet hedging and plasticity strategies.

We showed that, as in many other species, the environment can indeed have an influence on the chromatin structure of schistosomes (Roquis et al., 2014, 2016; Fneich et al., 2016) and epigenetic memory was identified as a promising drug target (Cabezas-Cruz et al., 2014). Besides this, since histones and histone modifications are extremely conserved through all taxa, histone methyltransferase inhibitors developed to treat human cancer have been used to understand specific functions for the lysine or arginine residues they modify in adult schistosomes (Padalino et al., 2018). Our group also demonstrated that histone deacetylation and demethylation inhibitors can reversibly inhibit miracidium to sporocyst transitions, suggesting that heterochromatization is important during this step (Azzi et al., 2009; Roquis et al., 2018). Our results indicate that HMT activity is essential for parasite development, and therefore, this class of enzymes represents a suitable drug target. It remains to be seen whether differences in the environment do indeed lead to heritable changes in one of the bearers of epigenetic information, such as histone modifications, DNA methylation, non-coding RNA or topology of the interphase nucleus in schistosomes.

## Conclusion

The way the inheritance system interacts with the environment could simply be of academic interest. However, the importance of this interaction becomes evident when considering how to design control measures. (Epi)Genome editing is, for the moment, out of reach, or only available in the laboratory. Control measures that influence epigenetics will, therefore, rely on changes to environmental cues. These changes could effect (I) selection of phenotypes, and (II) modifications in the inheritance system. Designing interventions that capitalize on a better understanding of epigenetic mechanisms in hosts and parasites poses a challenge since it can lead to unexpected and undesired outcomes, but also could represent a new opportunity: once we know how environmental cues trigger phenotypes, we might be able to push the right environmental “button” to effect lasting changes in schistosome infectivity and transmission.

## Author Contributions

RA contributed to the conceptualization, the investigation, the methodology, the project administration, the visualization, the writing of the original draft and the reviewing and editing of the manuscript. DD contributed to the conceptualization, the investigation, the methodology, the visualization, the writing of the original draft, and the reviewing and editing of the manuscript. CG contributed to the conceptualization, the investigation, the methodology, the funding acquisition, the visualization, the writing of the original draft, and the reviewing and editing of the manuscript.

## Conflict of Interest Statement

The authors declare that the research was conducted in the absence of any commercial or financial relationships that could be construed as a potential conflict of interest.
